# Exploring the known chemical space of the plant kingdom: insights into taxonomic patterns, knowledge gaps, and bioactive regions

**DOI:** 10.1186/s13321-023-00778-w

**Published:** 2023-11-10

**Authors:** Daniel Domingo-Fernández, Yojana Gadiya, Sarah Mubeen, David Healey, Bryan H. Norman, Viswa Colluru

**Affiliations:** Enveda Biosciences, Boulder, CO USA

**Keywords:** Drug discovery, Phytochemistry, Natural products, Chemotaxonomy

## Abstract

Plants are one of the primary sources of natural products for drug development. However, despite centuries of research, only a limited region of the phytochemical space has been studied. To understand the scope of what is explored versus unexplored in the phytochemical space, we begin by reconstructing the known chemical space of the plant kingdom, mapping the distribution of secondary metabolites, chemical classes, and plants traditionally used for medicinal purposes (i.e., medicinal plants) across various levels of the taxonomy. We identify hotspot taxonomic clades occupied by a large proportion of medicinal plants and characterized secondary metabolites, as well as clades requiring further characterization with regard to their chemical composition. In a complementary analysis, we build a chemotaxonomy which has a high level of concordance with the taxonomy at the genus level, highlighting the close relationship between chemical profiles and evolutionary relationships within the plant kingdom. Next, we delve into regions of the phytochemical space with known bioactivity that have been used in modern drug discovery. While we find that the vast majority of approved drugs from phytochemicals are derived from known medicinal plants, we also show that medicinal and non-medicinal plants do not occupy distinct regions of the known phytochemical landscape and their phytochemicals exhibit properties similar to bioactive compounds. Moreover, we also reveal that only a few thousand phytochemicals have been screened for bioactivity and that there are hundreds of known bioactive compounds present in both medicinal and non-medicinal plants, suggesting that non-medicinal plants also have potential therapeutic applications. Overall, these results support the hypothesis that there are many plants with medicinal properties awaiting discovery.

**Supplementary Information:**

The online version contains supplementary material available at 10.1186/s13321-023-00778-w.

## Introduction

Plants have routinely been a primary source of Natural Products (NPs) for drug development [1]. Traditionally, the choice of plants for investigation has been guided by ethnobotanical knowledge (i.e., the traditional use of plants by people of different cultures for various purposes, including medicinal), which has led to the discovery of myriads of potent bioactive compounds [2]. However, despite centuries of research, thousands of new plant species continue to be discovered each year, underscoring the immense diversity of plants and the complexity of their taxonomy. Only a limited region of the chemical space within the 300,000 known species of plants has been studied [3]. Furthermore, it is estimated that merely a fraction of the world's plant species have been tested for medicinal purposes [4]. Therefore, analyzing the chemical space of yet unexplored plants represents a promising avenue to discover new bioactive compounds and unlock the full medicinal potential of this diverse group of organisms.

Plants respond to their environments through the production and utilization of secondary metabolites [5]. Secondary metabolites are non-essential chemical compounds produced by living organisms with distinct roles in environmental interactions. Phytochemicals correspond to all plant-derived secondary metabolites, among which some exhibit biological effects (i.e., are bioactive) to, for example, help plants resist fungi, bacteria and plant virus infections. Secondary metabolites exhibit a vast and distinct chemical diversity that enables them to interact optimally with biological macromolecules, such as proteins [6]. Furthermore, secondary metabolites are often specific to taxonomic groups since they are typically produced by conserved metabolic routes [7, 8]. Thus, one approach to exploring the phytochemical space is to examine the distribution of secondary metabolites across various taxonomic groups. This can help identify unexplored or under-studied regions, making it an effective method for investigation.

To generate a chemical space, researchers can adopt either a knowledge-driven or data-driven approach. Knowledge-driven approaches involve integrating chemical and taxonomic information from published literature, either systematically [9] or by focusing on specific genera [10, 11] or families [12]. In contrast, data-driven approaches, such as those used by Defossez et al. [13] and Allard et al. [14], aim to represent the phytochemical space by characterizing the compounds present in plant extracts using techniques such as untargeted metabolomics. Although data-driven metabolomics approaches seek to systematically characterize all compounds in an extract and discover new chemicals, this task remains extremely difficult due to technical challenges, including the low annotation rates from MS2 spectra to structures [15] and the limited abundance of compounds in a given extract [16].

Once the chemical space has been defined using one of the two methods mentioned above, it is commonly analyzed in conjunction with a taxonomic or phylogenetic tree (see Defossez et al. [13] and Allard et al. [14], among others). It is also possible to directly classify and group species based on the similarities and/or differences in their chemical compositions. This classification method, known as a chemotaxonomy [17], has been used in several previous studies to classify specific families or genera based on their secondary metabolites [12, 18–20]. To our knowledge, a chemotaxonomic approach has not yet been systematically applied throughout the entire plant kingdom, which would not only allow for exploring the concordance between the known chemical space and the current taxonomy, but also for identifying regions covered by each taxon, potentially revealing knowledge gaps.

Here, we reconstruct the known chemical space of the plant kingdom to reveal the distribution of secondary metabolites, chemical classes, and medicinal plants. We then generate a comprehensive chemotaxonomy which we compare to a plant taxonomy, explore bioactive regions of the phytochemical space, and investigate differences in chemical properties and bioactivity between medicinal and non-medicinal plants. By exploring the chemical space through the lens of plant taxonomy, we identified taxonomic clades that require further characterization with regard to their chemical composition, as well as taxonomic hotspots occupied by a large proportion of medicinal plants and known secondary metabolites. In a complementary analysis, a chemotaxonomic approach of clustering plants based on their chemical profiles revealed a high degree of alignment with the taxonomy at the genus level. By studying the regions of the phytochemical space that are known to be bioactive and assessing their correspondence to the chemical space covered by approved drugs, we find that the majority of the approved drugs derived from phytochemicals are found in known medicinal plants with traditional medicinal usage. However, our in-depth analysis reveals that this observed prevalence cannot be explained by variances in the properties or bioactivity of the phytochemicals found in medicinal versus non-medicinal plants. Lastly, we shed light on the disproportionate emphasis placed on studying known medicinal plants, and highlight the existence of a wealth of untapped medicinal plants within the plant kingdom.

## Methods

### Datasets

#### Phytochemicals

As a proxy to represent the phytochemical space, we leveraged two of the most comprehensive NP databases: COCONUT [21] and LOTUS [9]. To normalize the chemical structures in both databases, we mapped the SMILES and InChIKeys of the SDF database dumps (version January 2022 and February 2022, respectively) to PubChem identifiers for each compound. Next, we matched their taxonomic information to NCBI Taxonomy identifiers [22] using fuzzy matching between the species name to species names or synonyms in the NCBI Taxonomy using the same procedure described in our previous publication [7]. After the normalization, we removed 262 compounds found in more than 100 plants, as they likely correspond to ubiquitous primary metabolites present in every plant. In total, the combined dataset contained 87,019 unique chemicals present in 19,987 plants. To construct a high-level map of the phytochemical space, we mapped the individual compounds to chemical classes using NPClassifier [23], similar to previous work [13, 14].

#### Approved drugs

We used three datasets listing approved drugs [2, 24], and FDA Approved Drug Products (Orange Book)). The first dataset by Wishart et al. [24] (v5.1.10) was obtained as an SDF file and contained the structures of 2289 approved drugs with a molecular weight greater than 150 Da. The second set of approved drugs was obtained from a dataset curated by Newman and Cragg, containing approved drugs from 1981 to 2019, and accompanying information on whether the drugs are derived from NPs or synthetic chemicals. As this dataset contained 1,881 trade names of approved drugs, many of which corresponded to the same compound, we first removed duplicates. Secondly, we filtered out 141 vaccines, 346 biological macromolecules (e.g., antibodies and therapeutic peptides), and other small structures with molecular weights less than 150 Da. Next, we automatically mapped drug names using the PubChem API and manually mapped the remaining ones. After this process, we obtained 1,291 unique structures, from which 375 are cataloged as NP-derived and 916 as synthetics. Additional file 1: Table S1 shows a comparison of the original and resulting dataset. Similarly, for the FDA Orange book, we mapped drug names with the PubChem API. Finally, for all three datasets, we matched their structures to phytochemicals using InChIKeys and resolved duplicates for drugs with multiple conformations.

#### Cataloging species as medicinal plants

Medicinal plants are species that have traditionally been used for medicinal purposes since they possess therapeutic properties or exert beneficial pharmacological effects on the human or animal body [25–27]. Given this broad definition, we leveraged a dataset derived from 33 million PubMed articles and ethnobotanical databases [7]. Overall, the dataset contains 97,066 plant-disease associations across 6,048 unique plants.

#### Bioactivity data

To analyze the bioactivity of the phytochemicals and the drug property space [28], we downloaded the SQL dump of the ChEMBL database (version 32) [29], a widely-established resource for molecules with drug-like physicochemical properties, and extracted all human bioassays with phytochemicals with either a direct effect on a target or an indirect effect via a cellular process. Following this, we categorized each bioassay into active and inactive based on its activity in the micromolar range using pChEMBL values (i.e. pChEMBL ≥ 6 representing bioactive compounds). In total, we extracted bioactivity information for 19,144 phytochemicals pertaining to 11,384 bioassays as well as metadata of the assay, such as the year.

### Establishing a chemotaxonomy of the plant kingdom

In this section, we outline our approach to defining a taxonomy based on chemical similarity. Our aim was to explore the chemical relatedness of a set of plants and compare the resulting clusters with taxonomic clades obtained from NCBITaxonomy [22]. To address the unbalanced distribution of chemicals and information across taxonomic clades, as well as the presence of promiscuous metabolites in the dataset, we established the following criteria: (i) exclusion of plants with less than 25 reported chemicals, (ii) exclusion of genera with less than five plants, and (iii) exclusion of chemicals present in more than 15 plants (Additional file 1: Fig. S1). These criteria enabled us to conduct the analysis focusing on plants with abundant chemical information, reduce the clustering to clades with enough species, and eliminate potentially biased results due to promiscuous chemicals, respectively.

To evaluate the chemical similarity of the 1017 plants belonging to 24 genera and 34 families that met our criteria, we constructed a similarity matrix by computing the Szymkiewicz-Simpson coefficient for each pair of plants based on the overlap of their respective sets of chemicals. We then transformed the similarity matrix into a distance matrix by subtracting 1 from each value. Using average linkage, we performed hierarchical clustering and generated the same number of clusters as the number of genera or families in all analyzed plants, depending on the level of the taxonomy evaluated. We used the adjusted Rand index to assess the agreement between the chemically-derived clusters and the taxonomic clades obtained from NCBITaxonomy.

### Comparing the chemical space of medicinal and non-medicinal plants at multiple levels: properties, scaffolds, and classes

We compared the chemical space of medicinal and non-medicinal plants at three distinct levels: (i) chemical properties, (ii) chemical scaffolds, and (iii) chemical classes. To calculate the chemical properties and Murcko scaffolds [30] of compounds, we used RDKit [31] (v2023.03.1). Similar to the previous section, we mapped individual compounds to their corresponding chemical classes using NPClassifier and generated a vector for each plant with the number of chemicals that belonged to each class.

While chemical classes of medicinal and non-medicinal plants could be directly calculated by computing class similarity between each plant pair and subsequently comparing the similarity between three distinct groups (i.e., pairs of medicinal plants, pairs of non-medicinal plants, and pairs of a medicinal plant and non-medicinal plant), the vectors of non-medicinal plants are significantly more sparse than the ones of medicinal plants. Furthermore, the similarity between pairs of plants would be influenced by their taxonomic group, as plants within the same family contain more similar chemical classes in contrast to plants across different families [7]. Thus, we decided to treat each plant family independently and divide each family into two groups (i.e., medicinal and non-medicinal plants). For each group, we generated a vector representing the number of compounds found in the 567 chemical classes. Next, we excluded plant families in which either group (i.e., medicinal or non-medicinal plants) covered less than ten chemical classes, reducing the number of plant families to 203 from a total of 365. Lastly, we normalized the vectors to represent the relative abundance of each chemical class by dividing the vector by the total number of compounds within the group.

We used t-SNE [32] to visualize the vectors and determine whether medicinal and non-medicinal plants could be accurately clustered. To further verify that there were no differences in the chemical classes between medicinal and non-medicinal plants, we trained an elastic net penalized logistic regression model [33] to predict the class label (i.e., medicinal and non-medicinal plants) on these vectors. We evaluated the performance through a fivefold stratified cross-validation using the area under the ROC curve.

## Results

This section begins by investigating the chemical space of the plant kingdom by examining the distribution of three different dimensions across various hierarchical levels of the taxonomy: (i) secondary metabolites, (ii) chemical classes, and (iii) medicinal plants. In the following subsection, we build a chemotaxonomy which we compare with the plant taxonomy and use this to identify areas where medicinal plants are overrepresented. Next, we explore regions of the phytochemical space that are known to have bioactivity and have been used in drug discovery. In the final subsection, we investigate whether the phytochemicals in known medicinal plants are different from the ones in non-medicinal ones.

### Mapping the phytochemical landscape reveals taxonomic bias and knowledge gaps

**Fig. 1:**
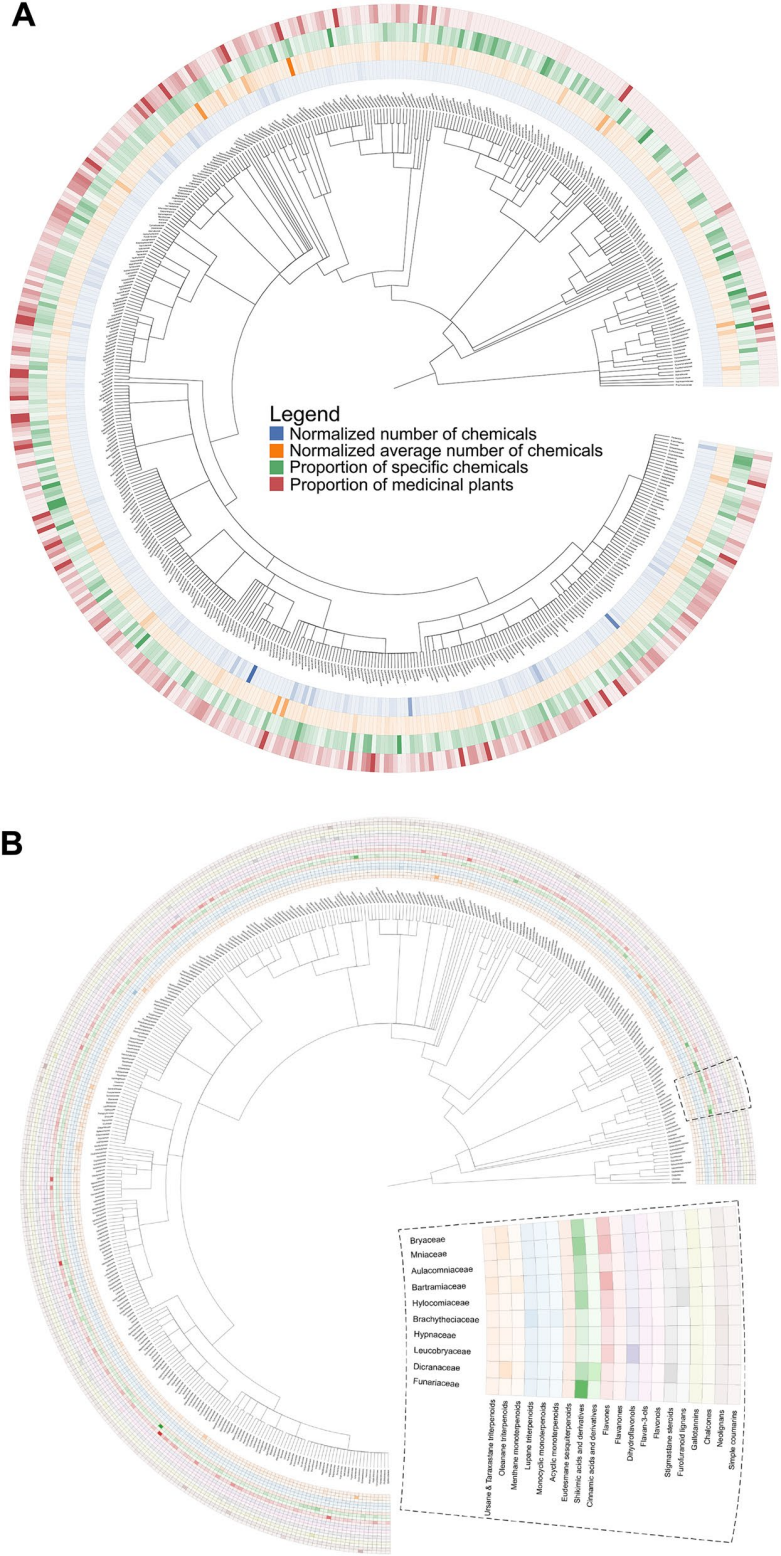
**A** Overview of the size and specificity of the chemical space across plant families. The blue column of the heatmap displays the normalized number of reported chemicals for each of the 513 families (i.e., leaf nodes in the phylogenetic tree). The red column represents the proportion of medicinal plants within the family. The green column highlights the proportion of phytochemicals that are unique to the family. Lastly, the orange column represents the average number of chemicals per species within the family. **B** Relative abundance of the 20 major secondary metabolite classes across plant families. Similar to (**A**) the leaf nodes in the phylogenetic tree correspond to different plant families. The heatmap indicates the relative abundance of each secondary metabolite class as a percentage with respect to the 567 chemical classes from NPClassifier [23]. Since the phylogenetic tree cannot be plotted with a heatmap with 567 columns (total number of chemical classes), we selected the 20 most abundant classes that were present in the majority of the plant families. Thus, only 319 of the 513 families which contained chemicals present in any of these 20 classes are depicted

We first explored the phytochemical space of different taxonomic clades to determine the extent of their chemical coverage and to identify any clades that were over- or under-investigated. Figure 1 depicts a phylogenetic tree illustrating the hierarchical relationships of taxa in the plant kingdom up to the family level. Additionally, the accompanying heatmap shows distinct properties of the chemical space across different plant families. Figure 1A (blue column) reveals differences in the number of reported phytochemicals for 513 families, which can vary from less than five (e.g., in Trimeniaceae and Asteliaceae) to several thousand (e.g., in Asteraceae, Fabaceae, and Lamiaceae). Despite these differences, generally, the number of phytochemicals was positively correlated with the number of species in a given plant family (Additional file 1: Fig. S2). We found that the distribution of the expected number of phytochemicals in a family (i.e., the average number of chemicals per plant multiplied by the number of species in the family) differed from the distribution of the observed number of phytochemicals (χ^2^ = 84,853, df = 512, *p* < 0.001) (Fig. 1A, orange column). Similarly, we observed the same trend at the genus level (χ^2^ = 40,5408, df = 4,843, *p* < 0.001), thus, confirming the presence of both overstudied and understudied taxonomic clades across the plant kingdom.

While thousands of phytochemicals have been reported for various families or genera, it is unclear whether these chemicals are unique to their particular taxonomic clades or if they are present across taxa. Thus, we inspected the number of chemicals specific to each family and genus (Fig. 1A, green column). Here again, we observed a positive correlation between the number of plants in a clade and the number of unique chemicals (Additional file 1: Fig. S2). Nonetheless, we also found discrepancies, as some clades tended to cover a broader unique chemical space than expected (χ^2^ = 28,627, df = 512, *p* ≤ 0.001). For instance, of the 2596 chemicals reported in the Myrtaceae family, 420 are specific to it. In contrast, other families with a comparable number of chemicals, such as Moraceae (2523), contain a much larger proportion of unique chemicals (1046).

These differences in chemical exclusivity among taxonomic clades raise some questions. Specifically, does this variation suggest that certain plant families produce a larger pool of secondary metabolites than others? Or, can we attribute these differences to our limited exploration of specific regions of the chemical space? Furthermore, are certain plant families enriched for medicinal plants, and if so, do they cover a broader phytochemical space compared to other plant families?

To investigate whether medicinal plants (i.e., plants associated with at least one indication in the scientific literature) possess any distinctive properties in the phytochemical space within the taxonomy, we determined their distribution across taxonomic clades. Analogous to the previous case, we found that larger families and genera tended to contain more medicinal plants (Fig. 1A, red column), however, medicinal plants were unevenly dispersed throughout the taxonomy (χ^2^ = 2,8627, df = 512, *p* = 2.0e−59). For instance, four of five plants with phytochemical information in the Saururaceae family are well-studied medicinal plants (e.g., *Saururus cernuus* [10, 11], *Houttuynia cordata* [34], *Anemopsis californica* [35]). In contrast, we found 220 families without any medicinal plants, despite some families having several dozens of plants (e.g., Restionaceae, Calceolariaceae, and Ancistrocladacea). Additionally, our analysis revealed that Marchantiophyta, also known as hepatics or liverworts, was the taxonomic group with the least number of medicinal plants (Fig. 1A; top right quadrant of heatmap), with only a few families containing species used for therapeutic applications.

Since these findings indicate that there is a subset of phytochemicals specific to each family, we next analyzed the distribution of different chemical classes of secondary metabolites across 319 families with abundant chemical information. The most prevalent classes among them were flavonols and steroids found in 305 and 290 families, respectively. Conversely, other classes, such as segatane and paraliane diterpenoids, were specific to certain taxonomic clades, like Euphorbia. Figure 1B displays a heatmap that shows the relative abundance of 20 of the major secondary metabolite classes in the 319 plant families. Overall, we observed a high degree of diversity, with some families abundant in certain chemical classes, and others abundant in altogether different ones. Revealing the distribution of specific phytochemicals across plant families is crucial as it informs us about the unique chemical profiles associated with different taxonomic groups.

### Clustering plants based on their chemical profile reconstructs the taxonomy at the genus level

**Fig. 2:**
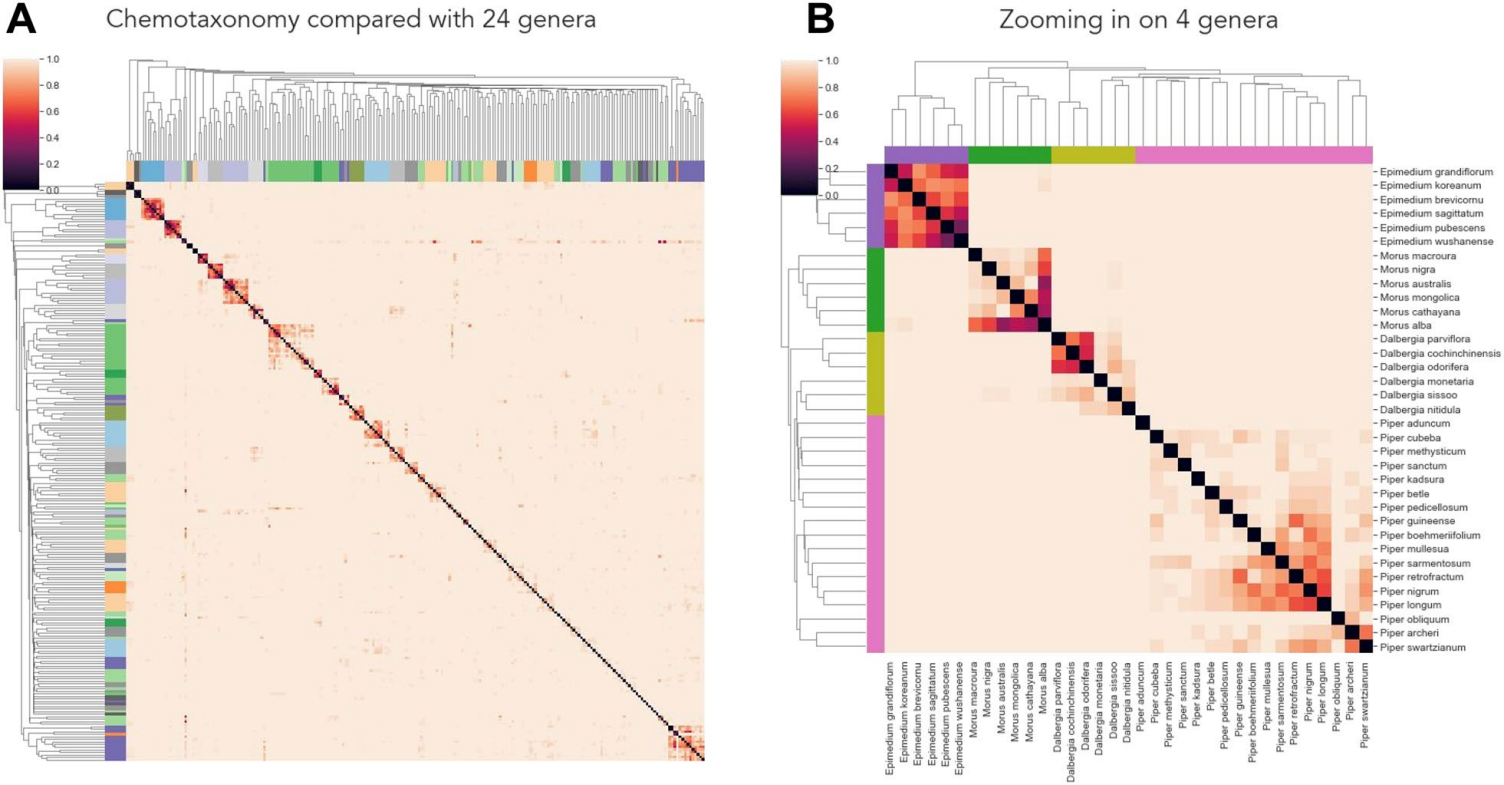
**A** Heatmap of the chemical similarity across the 24 largest genera based on number of plants and chemical information. The genus of each species is colored on the x and y axes. Note that the matrix displays the distance between pairs of species based on their chemical similarity. Details on the hierarchical clustering used and the definition of chemical similarity used to define the distance between the plants are described in the methods section. **B** Heatmap of chemical similarity focusing on a random subset of the 24 genera

Drawing from our observation that many chemicals and chemical classes are unique to specific taxonomic groups, we leveraged our chemotaxonomy to assess the concordance between the known phytochemical space and the taxonomy of the plant kingdom. To do so, we first clustered 1224 species found in 34 families based on their chemical similarity. Next, we calculated the agreement between the 34 clusters proposed by the chemotaxonomy and families of the plant taxonomy, resulting in an adjusted Rand index of 0.1. Given this relatively low value, we conducted a manual exploration of the chemotaxonomy, observing that, while some plant families were clustered correctly, others were combined into a single cluster due to their high chemical similarity. Thus, we repeated the clustering approach on a lower level of the taxonomy (i.e., genus) (Fig. 2A), finding that the agreement between the chemotaxonomy and the clades (i.e., genera) significantly improved (adjusted Rand index of 0.576). This suggests that, while the chemical profiles of plants may not accurately reflect family-level classifications, they can accurately classify plant species at a higher taxonomic resolution (i.e., genus). For example, the heatmap depicted in Fig. 2A revealed how most of the species of the same genus were accurately clustered, which can be attributed to a shared set of secondary metabolites within the clade. Moreover, by zooming into specific clusters (Fig. 2B), we verified how clustering plants based on their chemical profile can accurately recreate the taxonomic tree at the genus level.

Lastly, we investigated if there were clusters of plants from different genera enriched for medicinal plants, which would imply distinct areas of the chemical space for medicinal and non-medicinal plants. However, we did not find any such cluster with this approach that could distinguish plants with known therapeutic use from others.

### Medicinal plants are the main source of phytochemical-derived approved drugs

Having been unable to identify distinct areas of the chemotaxonomy occupied by medicinal versus non-medicinal plants, we sought to determine whether the number of approved drugs from NPs sourced from these two types of plants was roughly equivalent. Considering the greater emphasis on research dedicated to medicinal plants, we hypothesized that this group of plants would be over-represented amongst NP-approved drugs.

NPs and their derivatives can be obtained from various natural sources, such as plants, fungi, and microorganisms, and constitute over 30% of all approved drugs [2, 24]. We assessed the proportion of approved drugs from NPs that are derived from plants by overlaying phytochemicals with the dataset curated by Newman and Cragg [2] which catalogs compounds with annotations indicating whether the drug is NP-derived. Our analysis revealed that of the 167 approved drugs cataloged as NP-derived, 20.95% (35) are sourced specifically from plants (Additional file 1: Fig. S3A). These 35 compounds have been exclusively described in one or a few plant species, with the exception of humulene, which is present in extracts of numerous aromatic plants. Furthermore, to assess the coverage of the Newman and Cragg dataset, we overlaid phytochemicals with two additional lists of approved drugs [24], and FDA orange book), which yielded similar results (Additional file 1: Fig. S3B–D).

Finally, we determined whether approved drugs that originate from phytochemicals are derived from either medicinal or non-medicinal plants. Except for a small handful of phytochemicals present in more than five plants, such as humulene and artemisinin, we found that a disproportionate number of phytochemicals that are approved drugs are derived from known medicinal plants versus non-medicinal ones. Specifically, we found that 41 out of 47 plants containing these phytochemicals have been previously used in traditional medicine (Additional file 1: Table S2).

### Exploring the phytochemical space suggests that we are far from tapping the full therapeutic potential of plants

Prompted by our findings that a substantially larger proportion of NP-approved drugs are derived from medicinal plants, we further investigated whether the properties of phytochemicals in known medicinal plants are different from the ones in non-medicinal ones. Thus, we compared several chemical properties of the phytochemicals present in both groups, focusing on properties used to assess drug-likeness. These include molecular weight (MW), LogP, topological polar surface area (TPSA), and fraction of sp3 hybridized carbon atoms (Fsp3).

**Fig. 3:**
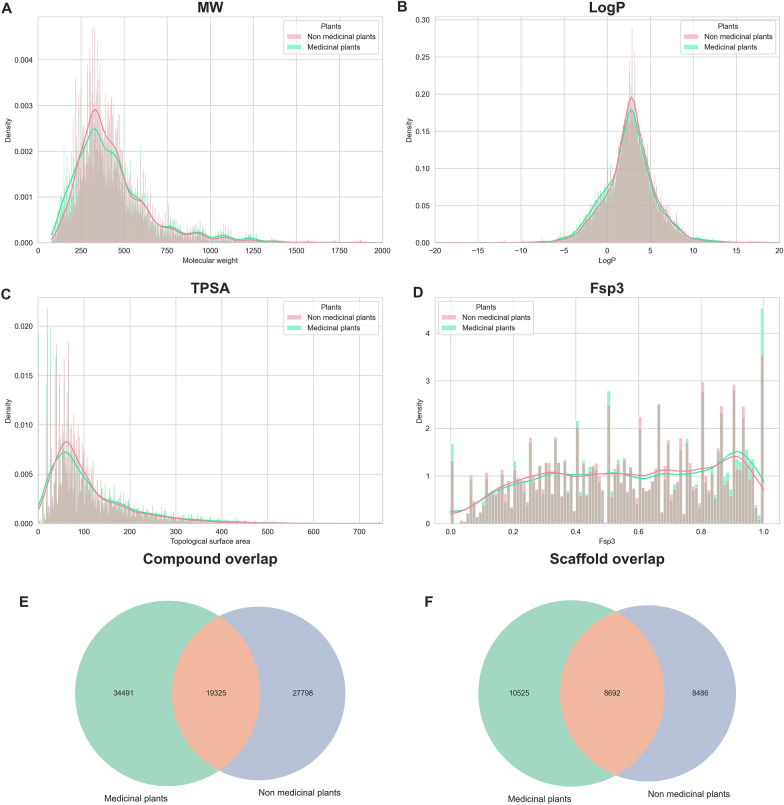
Distribution of the molecular weights (MW) (**A**), LogP (**B**), topological polar surface area (TPSA) (**C**), and fraction of sp3 hybridized carbon atoms (Fsp3) (**D**) of compounds in medicinal and non-medicinal plants. Overlap of compounds (**E**) and Murcko scaffolds (**F**) between medicinal and non-medicinal plants

Figure 3A–D revealed that both groups share analogous chemical properties, despite the relatively low overlap in compounds and scaffolds between them (Fig. 3E, F). Furthermore, we investigated whether there were differences in the chemical classes analyzed in "Mapping the phytochemical landscape reveals taxonomic bias and knowledge gaps" section (e.g., monoterpenoids, anthraquinones) for the compounds present in these two groups (i.e., medicinal and non-medicinal plants). A first inspection of the t-SNE visualization of the chemical classes for each family did not reveal any differences between medicinal and non-medicinal plants (Additional file 1: Fig. S4). Lastly, the performance of a machine learning classifier was close to random, indicating that there are no significant distinctions in chemical classes that differentiate medicinal from non-medicinal plants.

Given that medicinal plants have received far more attention in research [36], it is plausible that non-medicinal plants have simply been overlooked as a source of novel compounds with therapeutic potential. Thus, we performed an investigation to assess whether there are any differences in the bioactivity of phytochemicals between the two groups. To do so, we compared the same properties (i.e., MW, LogP, TPSA, Fsp3, compound overlap and scaffold overlap) for phytochemicals present in medicinal and non-medicinal plants to molecules with drug-like physicochemical properties in ChEMBL (Additional file 1: Fig. S5). Here, we found that both phytochemicals and ChEMBL compounds follow a similar distribution for all investigated properties, except for Fsp3, which is known to be higher in NPs [37]. We also confirmed that phytochemicals exhibited a greater diversity in terms of the relative number of unique scaffolds (35% vs. 31% for ChEMBL), as previously described by Yongye et al. [38] for NPs.

**Fig. 4:**
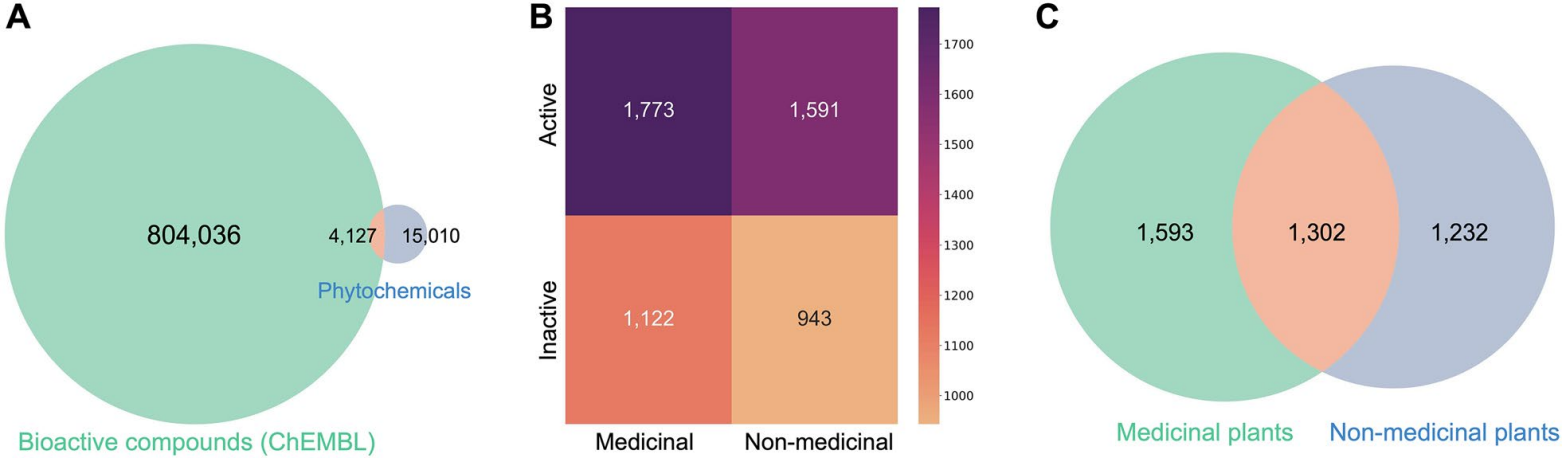
**A** Overlap between ChEMBL compounds with bioassay data and known phytochemicals mapped to ChEMBL (19,137 out of 87,019). Bioassay data represents the set of chemicals in ChEMBL whose bioactivity (active or inactive) has been evaluated. **B** Number of bioactive and non-bioactive compounds (represented as ‘active’ and ‘inactive’, respectively) in medicinal and non-medicinal plants. **C** Overlap of all bioactive compounds derived from medicinal and non-medicinal plants based on their bioassay information in ChEMBL

Next we leveraged the publicly curated bioassay data available in ChEMBL to examine the bioactivity of NPs. Notably, we found that only about 5% of all phytochemicals have reports of testing in biochemical or functional assays (Fig. 4A), indicating that only a small fraction of the phytochemical space has been screened for bioactivity. Furthermore, unlike approved drugs derived from phytochemicals, we did not find that bioactive phytochemicals were primarily derived from medicinal plants, since both medicinal and non-medicinal plants presented a similar number of bioactive phytochemicals (i.e., 1773 and 1591 respectively) (Fig. 4B). The slight difference in the number of bioactive compounds observed in medicinal versus non-medicinal plants can be attributed to a somewhat larger pool of phytochemicals in the former (44,158) compared to the latter group of plants (39,973). Despite the similar number of phytochemicals between the two groups, it is important to note the difference in the number of species, since there are reported to be three times as many non-medicinal plants as there are medicinal ones in our data (i.e., 15,545 versus 4257). This large difference highlights that non-medicinal plants have been significantly under-studied. Furthermore, we revealed that there are over 1300 known bioactive compounds present in non-medicinal plants (Fig. 4C), suggesting that we are far from cataloging all medicinal plants in the plant kingdom. One possible explanation for the difference in the number of approved drugs and bioactive phytochemicals derived from medicinal and non-medicinal plants is the time gap in which the data was collected. Most NP-derived drugs were discovered several decades ago with a focus on medicinal plants as these historical priors were considered good starting points for drug discovery [39]. For instance, in our dataset, 1994 was the average year of approval for drugs derived from phytochemicals, which means that the development of these drugs dates back to the 70 s and 80 s. However, when looking at bioactivity data, we found that the vast majority of data we used has been generated in the last decades (Additional file 1: Fig. S6), and therefore, it may be less biased towards medicinal plants (see limitations paragraph in the discussion).

Finally, we mapped these bioactive compounds back to the (chemo)taxonomic tree to identify possible bioactive hotspots (Additional file 1: Fig. S7). Consistent with our previous findings, we observed that known bioactive regions are unevenly distributed throughout the taxonomic tree (χ^2^ = 3272.97, df = 382, *p* < 0.001), once again suggesting that some taxonomic clades have been more explored than others. For example, the family Meliaceae (mahogany family) and the genus Hypericum appear to be bioactive hotspots given that they both contain a greater number of bioactive compounds than expected (Additional file 1: Fig. S8). On the other hand, despite containing a large number of chemicals, the family Woodsiaceae (cliff ferns) and the genus Magnolia have almost no known bioactive compounds and a low number of reported medicinal plants and phytochemicals, suggesting that these clades may have been under-studied for their bioactive potential.

## Discussion

In this work, we explore the chemical space of the plant kingdom by studying the distribution of three different facets (i.e., secondary metabolites, chemical classes, and medicinal plants) across various levels of the taxonomy to reveal well-studied regions and knowledge gaps across taxonomic clades. Furthermore, we develop a chemotaxonomy to (i) assess the concordance between the known phytochemical space and the plant taxonomy, and (ii) identify areas of the chemotaxonomy where medicinal plants are enriched. In doing so, we found large agreement at the genus level and no enrichment of medicinal plants, respectively. Finally, we explored regions of the phytochemical space that are known to have bioactivity and have been used in modern drug discovery, and investigated whether the phytochemicals in known medicinal plants are different from the ones in non-medicinal ones.

We acknowledge some limitations in our work that warrant further discussion. Firstly, the chemical space for NPs used in our analysis is incomplete, as we analyzed a small fraction of what is estimated to be present in plant sources [40]. To address this, we utilized the two largest publicly available resources for phytochemicals and species. These resources primarily focus on secondary metabolites, which are more suitable for our analysis compared to resources that mainly focus on primary metabolites across a limited number of plant species, such as PlantCyc. Furthermore, we evaluated the completeness of our dataset by comparing it to specific work that catalogs the chemical space of specific genera [10, 11], finding that our dataset captured a larger number of phytochemicals. Similarly, while we employed ChEMBL to explore the bioactivity of phytochemical space, alternative resources such as PubChem [41] and BindingDB [42] could also be used.

A further limitation of our work is the lack of a clear definition for drugs derived from NPs. To address this, we used the most conservative classification by excluding synthetic drugs with a NP pharmacophore as well as NP mimics from the dataset curated by Newman and Cragg [2]. Additionally, we encountered a similar limitation given the abstract definition of a medicinal plant. Thus, in our work, we defined a medicinal plant as a plant associated with at least one indication. Furthermore, it is important to note that there is no gold-standard dataset for approved drugs, as hinted at by the low overlap between the three approved drug datasets we have employed in this work (Additional file 1: Fig. S9), which suggests that neither covers the full spectrum of approved drugs. Lastly, we would like to note that the majority of data in ChEMBL is recent, and thus, the comparative analysis between ChEMBL and phytochemicals partially lacks historical data from pharma generated several decades ago. This implies that both the overlap between phytochemicals and ChEMBL (Fig. 4A) and the number of active/inactive compounds in both medicinal and non-medicinal plants (Fig. 4B) are likely to be larger than what we observed.

The findings of this study suggest several directions for future research that could significantly contribute to the field. Firstly, as we continue to uncover the vast phytochemical space and discover more secondary metabolites, future studies can utilize our proposed chemotaxonomy to evaluate its alignment with taxonomic clades. This could involve expanding the scope of analyses to include more plant species or secondary metabolites, as well as refining methodologies for determining taxonomic relationships (e.g., phylogenetic analysis). Secondly, we believe that, similar to Newman and Cragg [2], future work should periodically track the proportion of NPs among novel approved drugs in order to reassess the current influence and impact of NPs on drug discovery. Thirdly, specific environmental conditions that a plant is subject to, such as humidity, environmental stress, and altitude, can determine the pool of secondary metabolites the plant produces, many of which are a part of chemical classes with known therapeutic effects. Thus, by incorporating the influence of these environmental conditions into our chemotaxonomy, we could identify patterns that contribute to the emergence of various phytochemicals. Lastly, investigating the relationship between the evolutionary history of plant species and the distribution of phytochemicals across taxonomic groups could shed light on the mechanisms driving the diversification of secondary metabolism in plants.

## Conclusion

Two main conclusions can be drawn from this work. Firstly, medicinal and non-medicinal plants do not occupy disparate regions of the known phytochemical landscape. This finding is evidenced by the lack of enrichment of medicinal plants in specific parts of the taxonomic tree, and the absence of clusters of medicinal plants in the chemotaxonomy. Furthermore, both the similarity in chemical drug-like physicochemical properties and the number of bioactive compounds between the two groups lends support to this conclusion. Secondly, our findings suggest that more emphasis has been placed on the study of plants that have traditionally been used for medicinal purposes. This is evidenced by the fact that while medicinal plants are the main source of phytochemical-derived approved drugs, both medicinal and non-medicinal plants contain a comparable number of bioactive phytochemicals amongst molecules with drug-like physicochemical properties. Based on these conclusions, it can be hypothesized that there are likely many plants with medicinal properties that are still awaiting discovery.

## Scientific contribution

A comprehensive investigation of the phytochemical space, aiming to understand the distribution patterns of secondary metabolites, bioactive structures, and medicinal plants throughout the taxonomy of the plant kingdom.

### Supplementary Information

**Additional file 1: Table S1.** Comparison of the original Newman dataset and the dataset used in this work after normalization and filtering. **Table S2.** List of plants containing phytochemicals that are now approved-drugs. The last column indicates whether the plant is considered a medicinal plant (e.g., has been traditionally used to treat indications). **Figure S1. Number of species (plants) and their corresponding genera and families after each filtering step for the showcase of the chemotaxonomy**. **Figure S2. A**) Correlation between the observed chemicals specific to the family against the expected chemicals specific to the same family, corrected by the total number of species in the family. **B)** Correlation between the observed number of medicinal plants found in the family against the expected number of medicinal plants belonging to the same family, corrected by the total number of species in the family. **C)** Correlation between the observed number of chemicals found in the family against the expected number of chemicals belonging to the same family, corrected by the total number of species in the family. The y-axis range is set to 1,000, although a few families have over 2,000 chemicals. **Figure S3. A**) Proportion of plant-specific compounds present in the NP approved-drugs curated by Newman and Cragg [2]. **B)** Overlap of the matching phytochemicals of the two datasets: Newman and Cragg [2] and Wishart et al. [24]. **C**) Number of plant-specific compounds present in the dataset curated by Wishart et al. [24]. **D**) Number of plant-specific compounds present in the FDA orange book*.* Figure 4**. t-SNE of the relative abundance of the chemical classes from NP-classifier. Figure S5. Distribution of four chemical properties between ChEMBL compounds (version 32) and all phytochemicals in medicinal and non-medicinal plants used in our work.** A) Distribution of the molecular weights (MW), B) Distribution of the LogP. C) Distribution of the topological polar surface area (TPSA) D) Distribution of the fraction of sp3 hybridized carbon atoms (Fsp3). **Figure S6. Comparison of the log distributions of NP approved drugs versus phytochemicals and their corresponding bioassays over time.** The plots highlight a decline in NP approved drugs around the late 1980s. At the same time, the plots indicate an increase in the number of phytochemicals being tested for bioactivity, especially in the last 15 years. **Figure S7. Distribution of the number of bioactive compounds in medicinal (inner circle) and non-medicinal (outer circle) plants across plant families.** Due to a few families having a disproportionately large number of bioactive compounds compared to the rest, we set their values to white any family with more than 150 bioactive compounds in both medicinal and non-medicinal plants to able to spot easier the differences between the two groups for the rest with a smaller range in the color palette (intensity). **Figure S8. Distribution of the number of observed and expected bioactive compounds across the plant families with the highest expected values.** The expected number of bioactive compounds is calculated by multiplying the average number of bioactive compounds per plant by the number of species in a family. The plot shows that a very low percentage of bioactive compounds have been identified in plant families such as Asreraceae and Fabaceae, unlike Pinaceae where the number of bioactive compounds identified is relatively similar to the number expected for this plant family. **Figure S9. Overlap between the DrugBank, FDA orange book and Newman and Cragg datasets**.

## Data Availability

Source code, raw and processed data, and Jupyter notebooks to reproduce the analyses are available at https://github.com/enveda/plant-chemical-space.
