# Supplementary material for: Exploring the known chemical space of the plant kingdom: insights into taxonomic patterns, knowledge gaps, and bioactive regions
Source: J Cheminform. 2023 Nov 10;15:107. doi: 10.1186/s13321-023-00778-w (PMC10636812; doi:10.1186/s13321-023-00778-w)
Supplement: Supplementary file 1 — Additional file 1: Table S1. Comparison of the original Newman dataset and the dataset used in this work after normalization and filtering. Table S2. List of plants containing phytochemicals that are now approved-drugs. The last column indicates whether the plant is considered a medicinal plant (e.g., has been traditionally used to treat indications). Figure S1. Number of species (plants) and their corresponding genera and families after each filtering step for the showcase of the chemotaxonomy. Figure S2. A) Correlation between the observed chemicals specific to the family against the expected chemicals specific to the same family, corrected by the total number of species in the family. B) Correlation between the observed number of medicinal plants found in the family against the expected number of medicinal plants belonging to the same family, corrected by the total number of species in the family. C) Correlation between the observed number of chemicals found in the family against the expected number of chemicals belonging to the same family, corrected by the total number of species in the family. The y-axis range is set to 1,000, although a few families have over 2,000 chemicals. Figure S3. A) Proportion of plant-specific compounds present in the NP approved-drugs curated by Newman and Cragg [2]. B) Overlap of the matching phytochemicals of the two datasets: Newman and Cragg [2] and Wishart et al. [24]. C) Number of plant-specific compounds present in the dataset curated by Wishart et al. [24]. D) Number of plant-specific compounds present in the FDA orange book. Figure 4. t-SNE of the relative abundance of the chemical classes from NP-classifier. Figure S5. Distribution of four chemical properties between ChEMBL compounds (version 32) and all phytochemicals in medicinal and non-medicinal plants used in our work. A) Distribution of the molecular weights (MW), B) Distribution of the LogP. C) Distribution of the topological polar surface area (TPSA) D) Distribution [file 13321_2023_778_MOESM1_ESM.pdf]

Additional File

Additional file Tables

Code (Newman)	Definition (Newman dataset)	Category	Number of rows in original file	Curated and mapped unique structures
N	unaltered natural product, 1997	NP	71	62
NB	botanical drug (defined mixture), 2012	NP	14	3
ND	natural product derivative, 1997	NP	356	333
S	synthetic drug, 1997	Synthetic	463	452
S*	synthetic drug (NP pharmacophore), 1997	Synthetic	65	61
/NM	mimic of natural product, 2003	Synthetic	207 (S*/NM) + 217 (S/NM)	205 (S*/NM) + 217 (S/NM)

Additional file 1: Table S1. Comparison of the original Newman dataset and the dataset used in this work after normalization and filtering.

NCBITaxonomy ID	Name	Medicinal plant? (use in traditional medicine)
ncbitaxon:46220	*Taxus brevifolia*	Yes
ncbitaxon:48119	*Glehnia littoralis*	Yes
ncbitaxon:405945	*Leonurus sibiricus*	Yes
ncbitaxon:431156	*Croton stellatopilosus*	Yes
ncbitaxon:555479	*Nigella sativa*	Yes
ncbitaxon:4682	*Allium sativum*	Yes
ncbitaxon:108594	*Campsis grandiflora*	Yes
ncbitaxon:296036	*Phyllanthus emblica*	Yes
ncbitaxon:147273	*Taxus wallichiana*	Yes
ncbitaxon:4058	*Catharanthus roseus*	Yes
ncbitaxon:191701	*Cephalotaxus hainanensis*	Yes
ncbitaxon:99806	*Taxus cuspidata*	Yes
ncbitaxon:66169	*Cephalotaxus fortunei*	Yes

ncbitaxon:65561	*Hypericum perforatum*	Yes
ncbitaxon:25629	*Taxus baccata*	Yes
ncbitaxon:109792	*Citrus natsudaidai*	Yes
ncbitaxon:376254	*Ranunculus ternatus*	Yes
ncbitaxon:2711	*Citrus sinensis*	Yes
ncbitaxon:43166	*Citrus aurantium*	Yes
ncbitaxon:154990	*Euphorbia helioscopia*	Yes
ncbitaxon:58029	*Cephalotaxus harringtonia*	Yes
ncbitaxon:3483	*Cannabis sativa*	Yes
ncbitaxon:330167	*Dioscorea villosa*	Yes
ncbitaxon:93608	*Sinopodophyllum hexandrum*	Yes
ncbitaxon:4227	*Flaveria trinervia*	Yes
ncbitaxon:4182	*Sesamum indicum*	Yes
ncbitaxon:329759	*Solanum aculeastrum*	Yes
ncbitaxon:35933	*Podophyllum peltatum*	Yes
ncbitaxon:29780	*Mangifera indica*	Yes
ncbitaxon:246360	*Commiphora wightii*	Yes
ncbitaxon:126910	*Withania somnifera*	Yes
ncbitaxon:4072	*Capsicum annuum*	Yes
ncbitaxon:137221	*Rheum palmatum*	Yes
ncbitaxon:37690	*Citrus trifoliata*	Yes
ncbitaxon:3993	*Euphorbia esula*	Yes
ncbitaxon:39354	*Salvia abrotanoides*	Yes
ncbitaxon:224740	*Juniperus sabina*	Yes
ncbitaxon:212925	*Euphorbia lathyris*	Yes
ncbitaxon:107238	*Croton sublyratus*	Yes
ncbitaxon:2067815	*Commiphora mukul*	Yes
ncbitaxon:88032	*Taxus canadensis*	Yes
ncbitaxon:85957	*Taxus x media*	No
ncbitaxon:2708766	*Cola ballayi*	No
ncbitaxon:69918	*Micranthemum umbrosum*	No
ncbitaxon:89484	*Cephalotaxus sinensis*	No
ncbitaxon:417013	*Livistoneae incertae sedis*	No
ncbitaxon:1721085	*Pentzia eenii*	No

Additional fil 1: Table S2. List of plants containing phytochemicals that are now approved-drugs. The last column indicates whether the plant is considered a medicinal plant (e.g., has been traditionally used to treat indications).

Additional file Figures

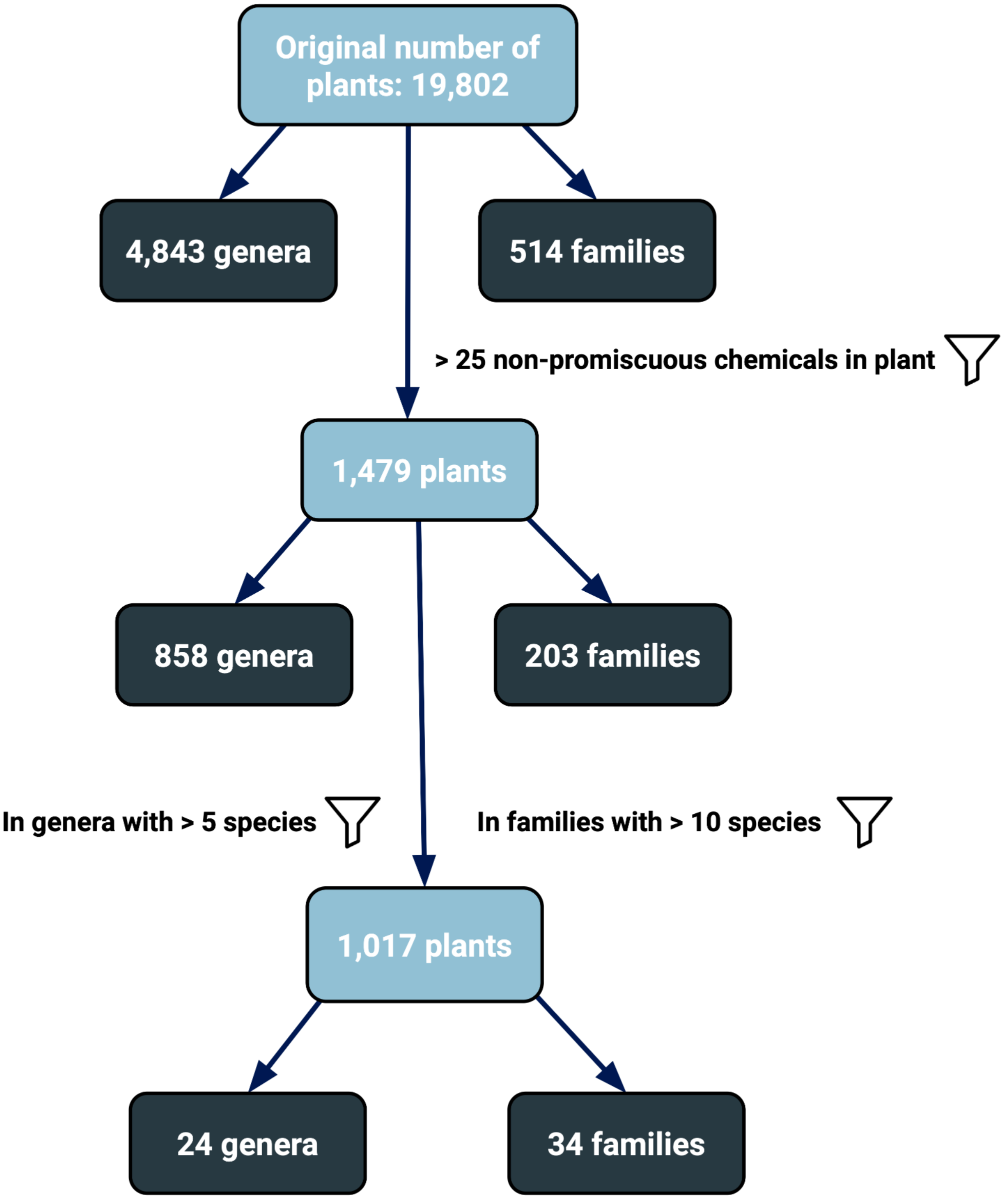

Additional file 1: Figure S1. Number of species (plants) and their corresponding genera and families after each filtering step for the showcase of the chemotaxonomy.

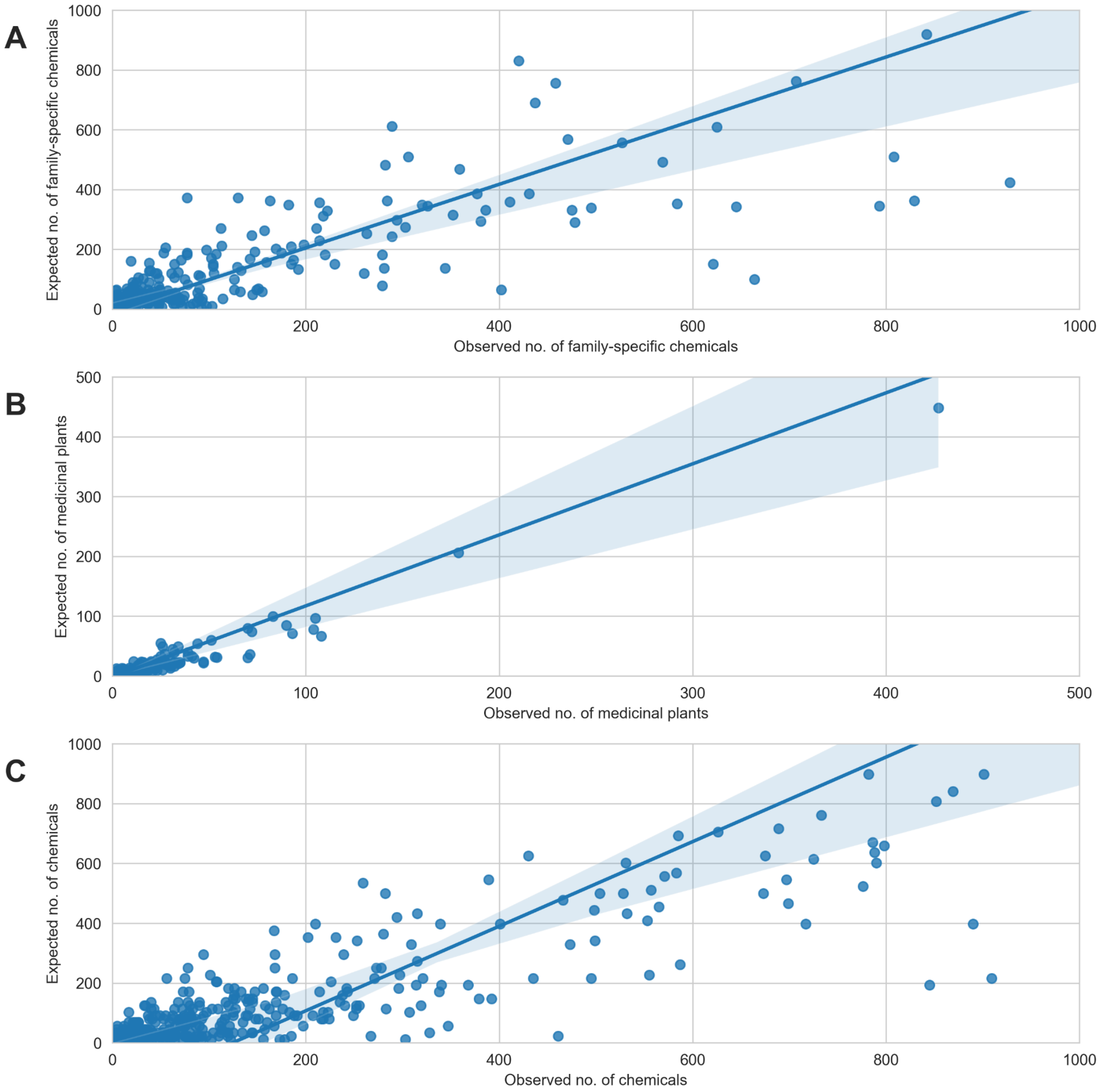

Additional file 1: Figure S2. A) Correlation between the observed chemicals specific to the family against the expected chemicals specific to the same family, corrected by the total number of species in the family. **B)** Correlation between the observed number of medicinal plants found in the family against the expected number of medicinal plants belonging to the same family, corrected by the total number of species in the family. **C)** Correlation between the observed number of chemicals found in the family against the expected number of chemicals belonging to the same family, corrected by the total number of species in the family. The y-axis range is set to 1,000, although a few families have over 2,000 chemicals.

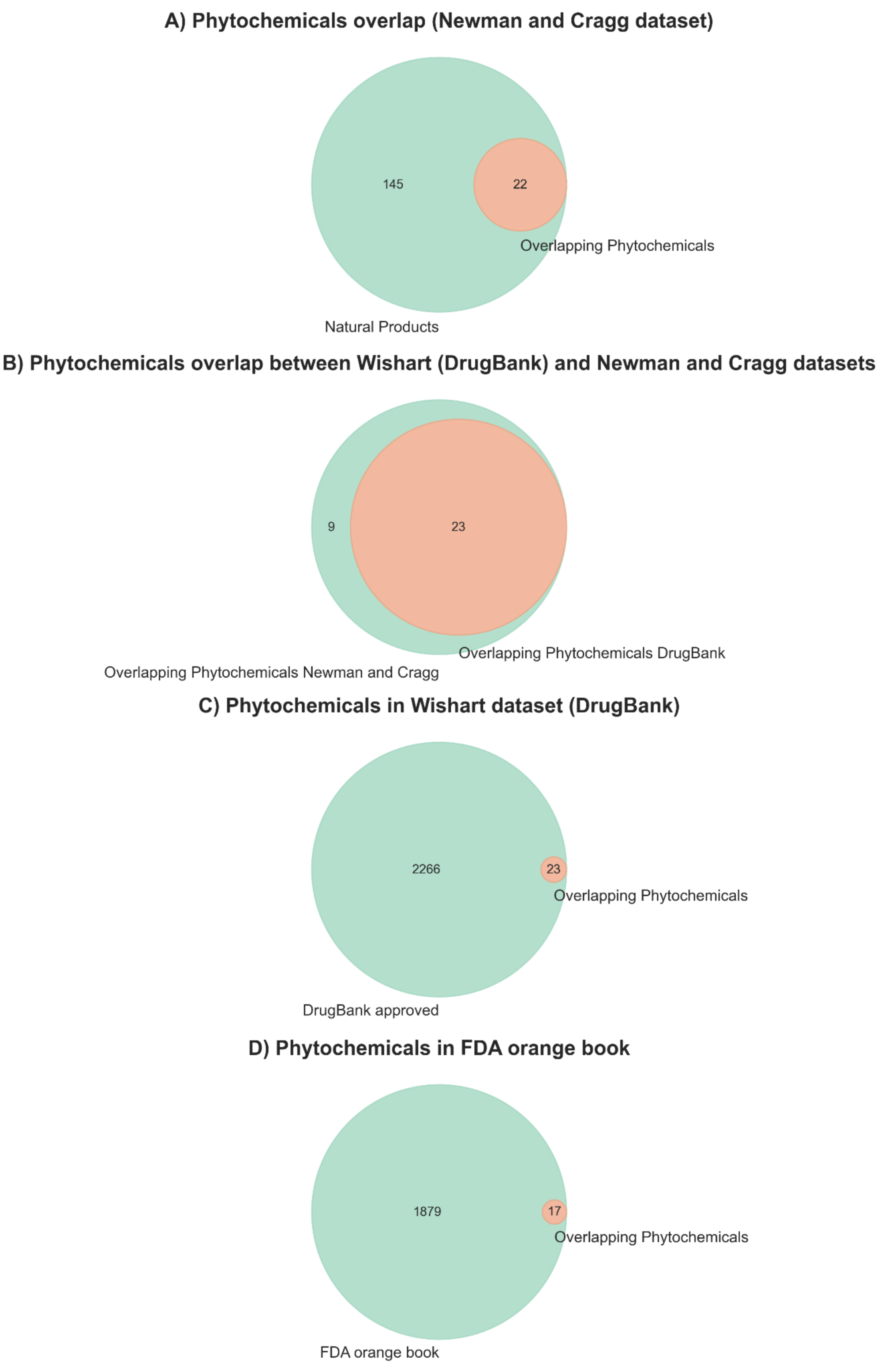

Additional file 1: Figure S3. A) Proportion of plant-specific compounds present in the NP approved-drugs curated by Newman and Cragg (2020). **B)** Overlap of the matching phytochemicals of the two datasets: Newman and Cragg (2020) and Wishart *et al.* (2018). **C**) Number of plant-specific compounds present in the dataset curated by Wishart *et al.* (2018). **D**) Number of plant-specific compounds present in the FDA orange book.

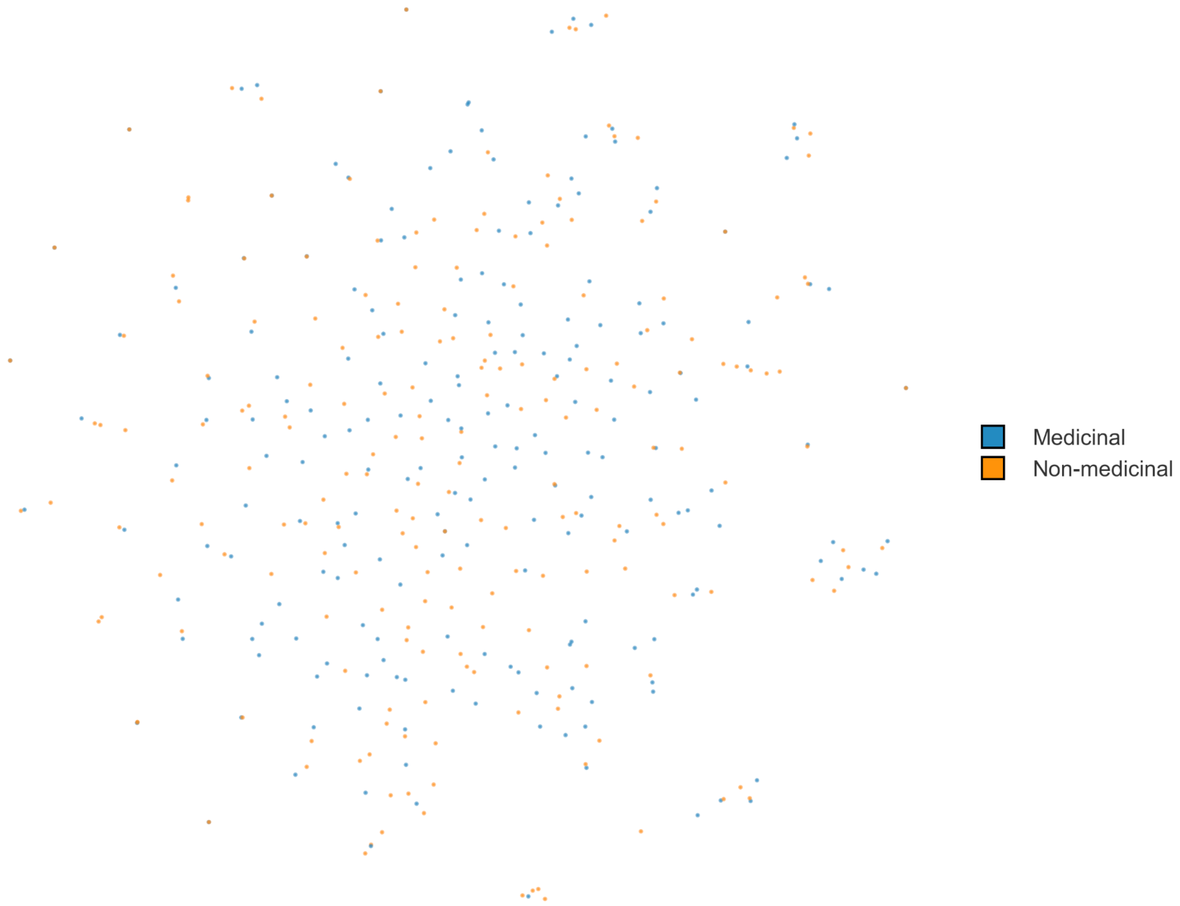

Additional file 1: Figure S4. t-SNE of the relative abundance of the chemical classes from NP-classifier.

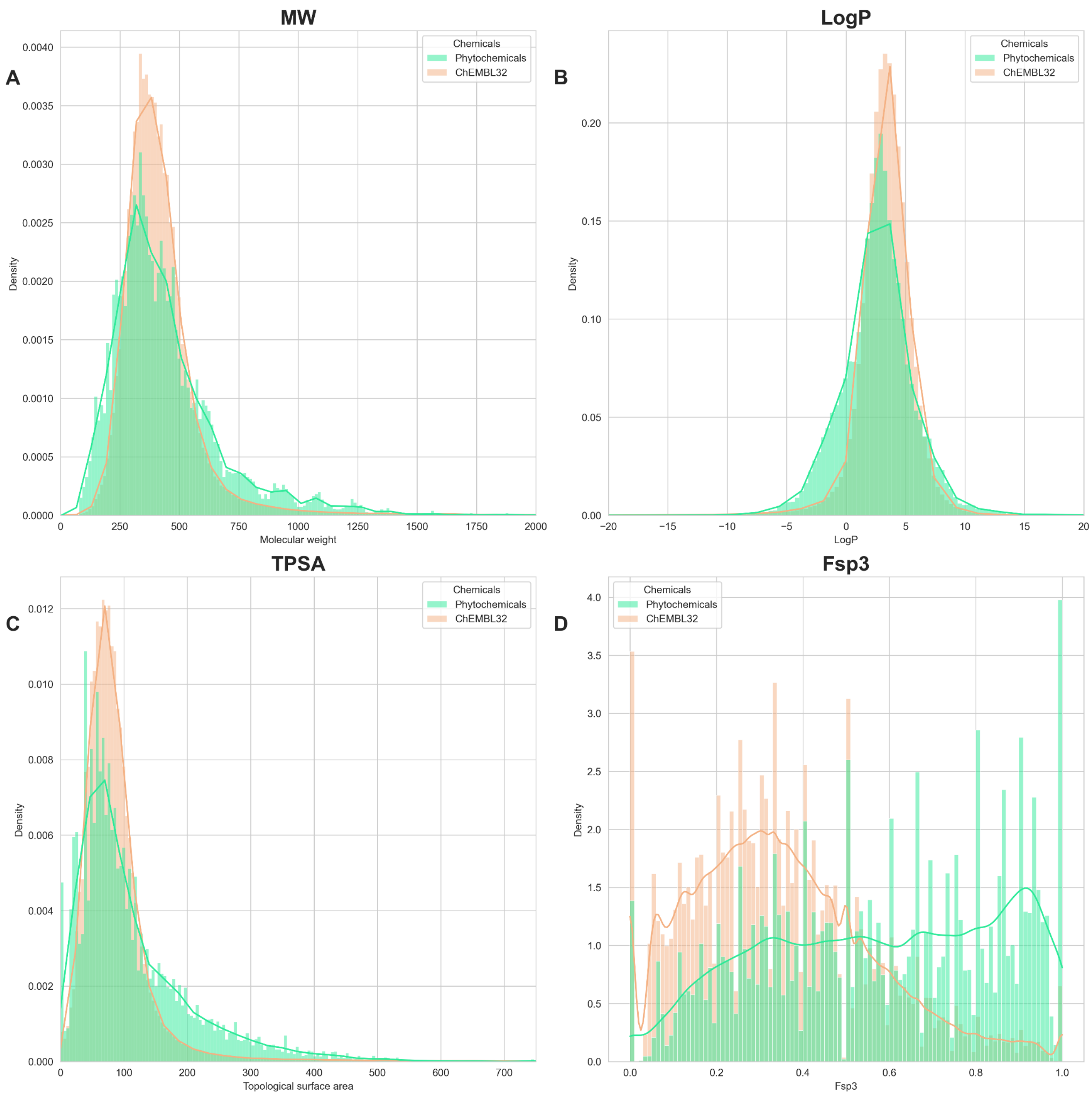

Additional file 1: Figure S5. Distribution of four chemical properties between ChEMBL compounds (version 32) and all phytochemicals in medicinal and non-medicinal plants used in our work. A) Distribution of the molecular weights (MW), B) Distribution of the LogP. C) Distribution of the topological polar surface area (TPSA) D) Distribution of the fraction of sp3 hybridized carbon atoms (Fsp3).

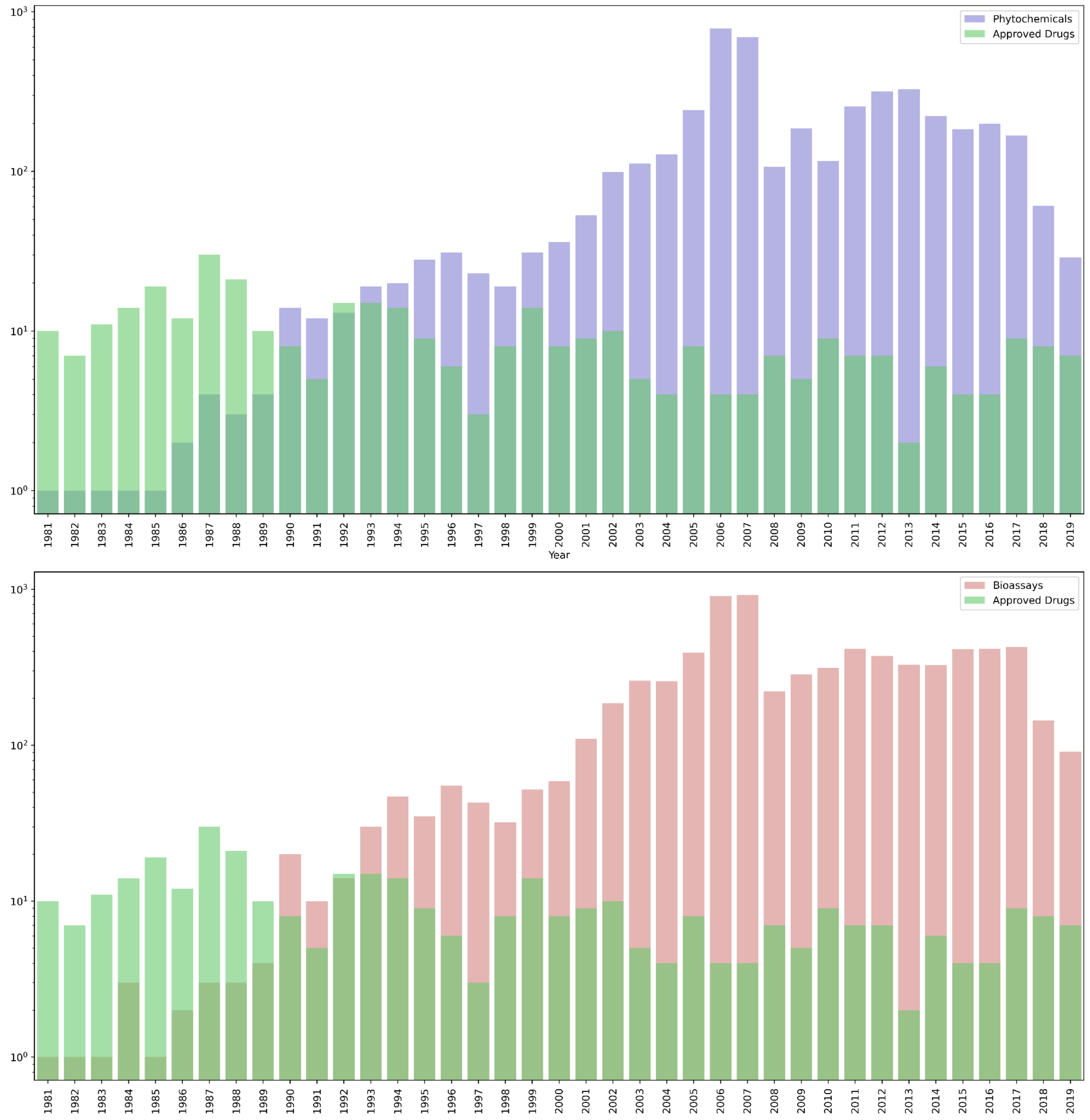

Additional file 1: Figure S6. Comparison of the log distributions of NP approved drugs versus phytochemicals and their corresponding bioassays over time. The plots highlight a decline in NP approved drugs around the late 1980s. At the same time, the plots indicate an increase in the number of phytochemicals being tested for bioactivity, especially in the last 15 years.

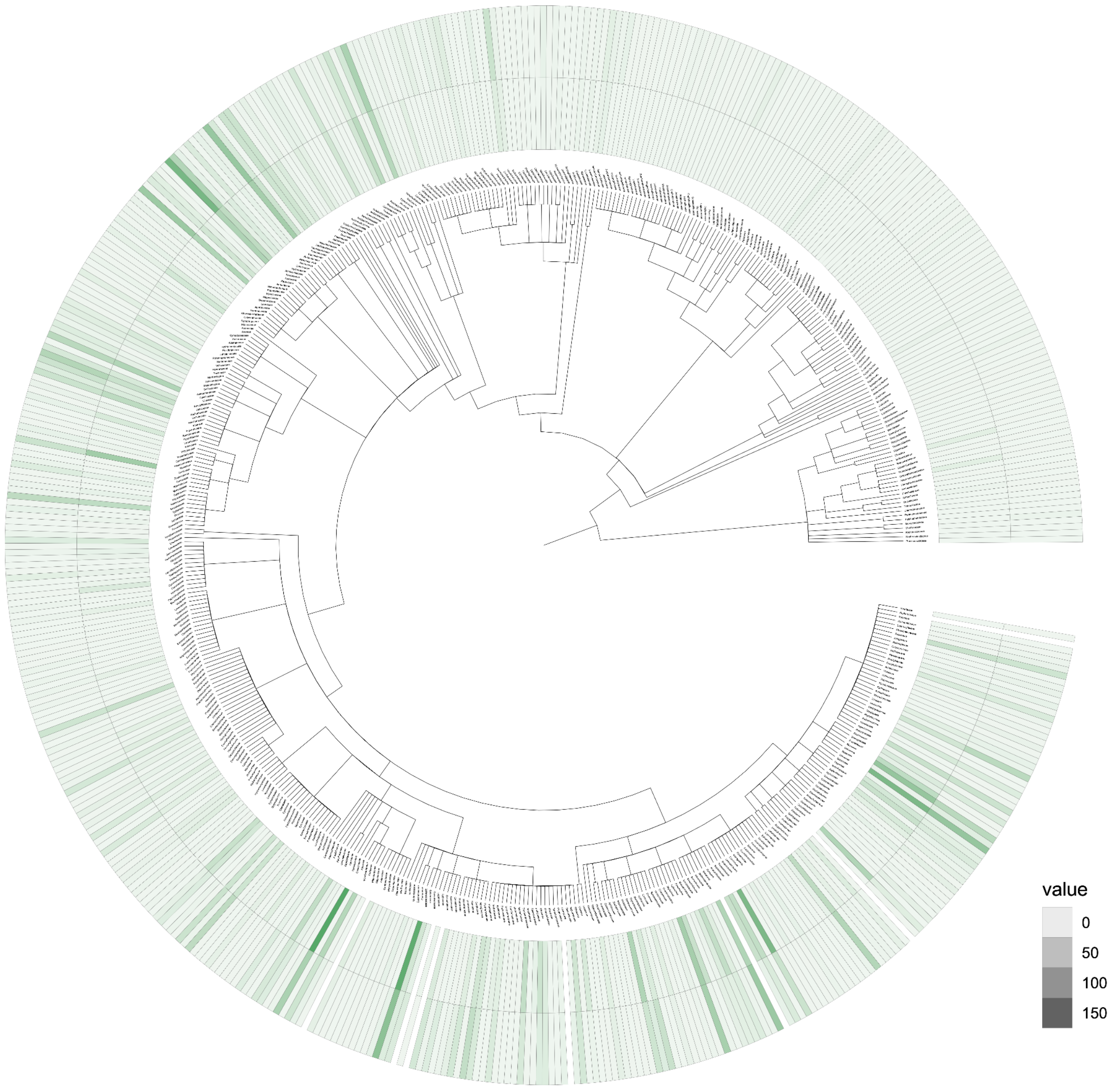

Additional file 1: Figure S7. Distribution of the number of bioactive compounds in medicinal (inner circle) and non-medicinal (outer circle) plants across plant families. Due to a few families having a disproportionately large number of bioactive compounds compared to the rest, we set their values to white any family with more than 150 bioactive compounds in both medicinal and non-medicinal plants to able to spot easier the differences between the two groups for the rest with a smaller range in the color palette (intensity).

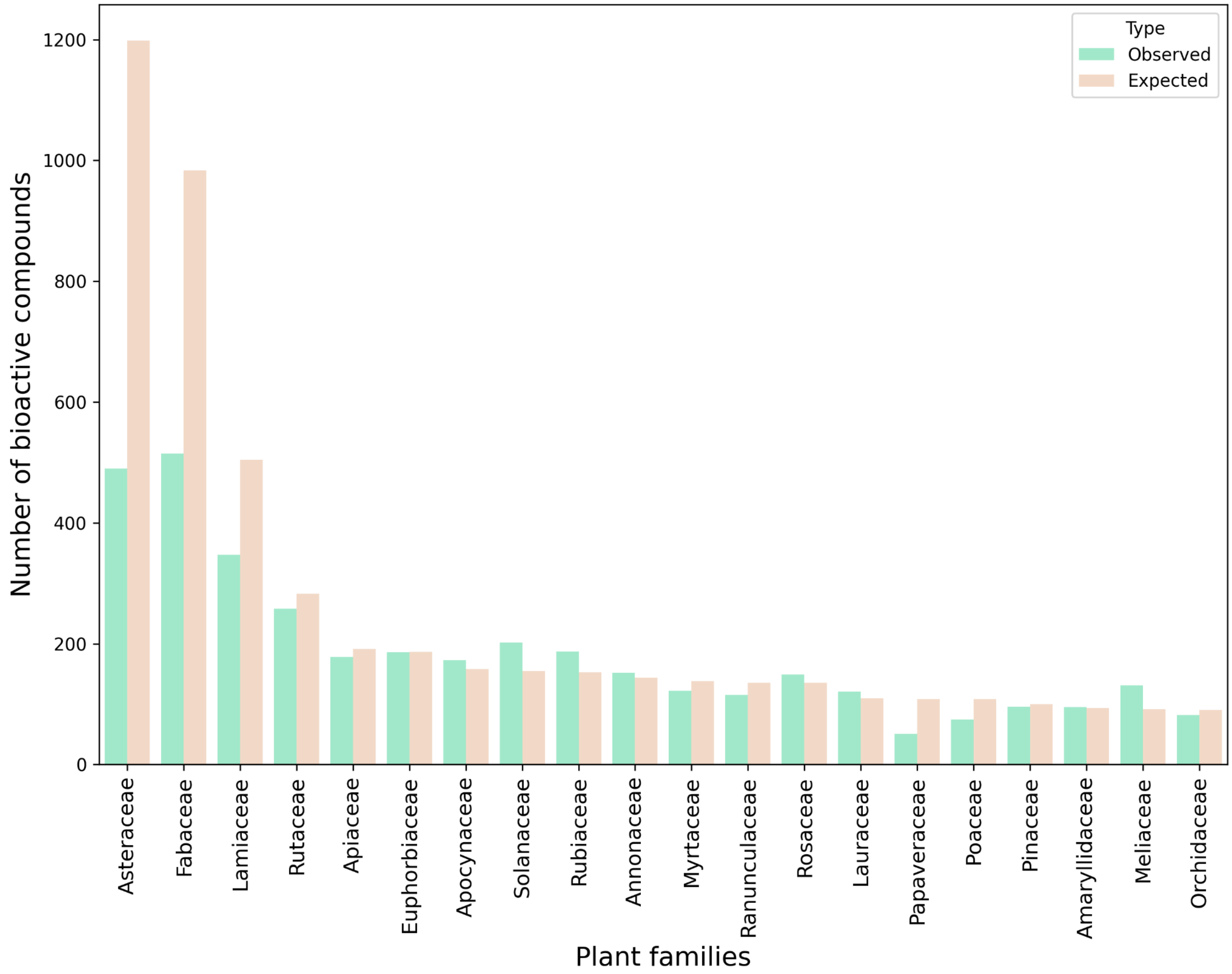

Additional file 1: Figure S8. Distribution of the number of observed and expected bioactive compounds across the plant families with the highest expected values. The expected number of bioactive compounds is calculated by multiplying the average number of bioactive compounds per plant by the number of species in a family. The plot shows that a very low percentage of bioactive compounds have been identified in plant families such as Asreraceae and Fabaceae, unlike Pinaceae where the number of bioactive compounds identified is relatively similar to the number expected for this plant family.

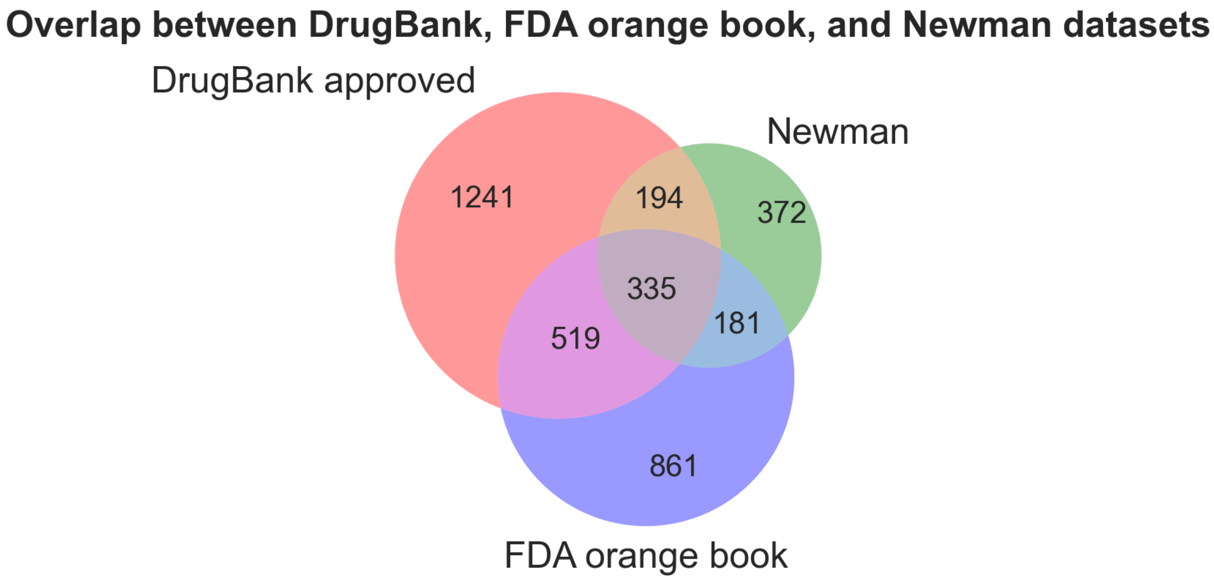

Additional file 1: Figure S9. Overlap between the DrugBank, FDA orange book and Newman and Cragg datasets.
